# Supplementary material for: Are carbon emissions trading and green financial instruments synergistic? -Comprehensive quantitative research based on content analysis
Source: PLoS One. 2024 Mar 7;19(3):e0298601. doi: 10.1371/journal.pone.0298601 (PMC10919601; doi:10.1371/journal.pone.0298601)
Supplement: S1 Data — (ZIP) [file pone.0298601.s001.zip › Data/地级市Z_F工作报告.zip!/─■╧─/╣╠╘¡╩╨/└┤╫╘╣1⁄2╓┌║┼í╛┬φ┐╦╩2╛▌═°í┐_2011.docx]

各位代表：

　　现在，我代表市人民政府向大会作政府工作报告，请各位代表连同已提交大会的《固原市国民经济和社会发展第十二个五年规划纲要(草案)》一并审议，并请市政协委员和列席人员提出意见。

　　2010年及“十一五”工作回顾

　　2010年，是我市应对各种挑战、全面推进经济发展战略转型、促进经济社会又好又快发展的一年，也是胜利实现“十一五”规划目标的收官之年。一年来，在自治区党委、政府和市委的正确领导下，全市广大干部群众以科学发展观为指导，围绕加快实现以农业为主导向以工业为主导、多产业发展并举转变的总体要求和“一五五”工作思路，提振信心，奋力攻坚，全力抓好事关经济社会发展的15件大事和改善民生的25件实事，圆满完成了市二届人大三次会议确定的各项目标任务及“十一五”规划目标。

　　一、以加快发展为主题，综合实力大幅提升

　　2010年，预计全市地区生产总值和全社会固定资产投资分别达到104亿元、112亿元，同比分别增长12.6%和30%，突破“两个百亿元”大关目标;地区生产总值是2005年2.32倍，年均增长12.6%;人均地区生产总值达到6846元(约1031美元)，同比增长16.9%，是2005年2.3倍，年均增长18%。“十一五”完成全社会固定资产投资338亿元，是“十五”的3倍，年均增长26%。地方财政一般预算收入达到5.2亿元，同比增长49%，是2005年3.7倍，年均增长30%。城镇居民人均可支配收入达到12973元，同比增长10%，比2005年增长98.7%。农民人均纯收入达到3420元，同比增长15.5%，比2005年增长99.4%。人口自然增长率控制在11.81‰以内，城镇登记失业率控制在4.2%以内，万元GDP能耗下降1.61%。

　　二、以结构调整为主线，三次产业快速发展

　　农业经济稳步发展。2010年，预计完成农业增加值28.9亿元，同比增长10%，是2005年2.25倍，年均增长8.4%。粮食总产量7.46亿公斤，创历史新高，比2005年增加1.41亿公斤。特色优势产业发展壮大，建成肉牛示范村105个，肉牛饲养量达到78万头，是2005年1.72倍;“泾源黄牛”通过地理标志认证。马铃薯种植面积218.6万亩，比2005年增加95.5万亩，总产201.1万吨，三级脱毒种薯繁育推广体系初步形成，建立脱毒种薯繁育基地14.45万亩，生产原原种6249万粒;“西吉马铃薯”荣获中国驰名商标。新增设施农业6.3万亩，累计达到22万亩;完成以覆膜保墒为主的旱作节水农业95.3万亩。原州蔬菜、西吉西芹、隆德花卉、泾源苗木、彭阳辣椒等新的特色产业正在形成。建立健全土地流转机制，积极推广西吉“华林模式”，引进总投资5000万元以上农业产业化龙头企业9家，“六盘山”牌农产品商标荣获自治区著名商标。

　　工业经济活力增强。2010年，预计完成工业增加值13.9亿元，同比增长25%，占GDP比重由2005年的10.6%提高到13.3%，年均增长17.3%。固原盐化工循环经济扶贫示范区启动建设，投资1.1亿元完成示范区总体规划、选址和10平方公里土地征用、主干道12.5公里路基及道路绿化。固原经济开发区中小企业科技创业园建成投入使用。六盘山热电厂2×330兆瓦机组并网发电，王洼二矿年产150万吨原煤项目建成试生产，银洞沟煤矿年产300万吨技改扩建、华电西吉月亮山风电厂一期49.5兆瓦等重点项目开工建设。彭阳石油资源开发进一步加快，新打油井30口，生产原油13.5万吨。全年生产原煤140万吨、水泥91.2万吨，分别是2005年2.46倍和2.14倍，能源工业迈出具有里程碑意义的一步。马铃薯、牛羊肉等农副产品加工业效益明显提高。深入实施“项目建设和招商引资年”活动，引进资金29.27亿元，同比增长88.2%;“十一五”累计引进资金76.4亿元，是“十五”的5.4倍。认真落实节能减排措施，化学需氧量和二氧化硫排放量分别控制在1万吨和0.98万吨以内，超额完成 “十一五”节能减排任务。

　　第三产业快速发展。2010年，预计完成三产增加值49.2亿元，同比增长10%，比2005年增加25.88亿元，年均增长12.8%。完成须弥山博物馆、六盘山生态博物馆、萧关遗址文化园等重大旅游基础设施建设和6条景区主干道宽幅绿化。“十一五”期间，六盘山红军长征纪念馆、将台堡红军长征纪念园建成使用，六盘山旅游区、固原博物馆被评为国家4A级景区，成功举办了五届六盘山山花旅游节等系列活动，累计接待游客547.15万人次，实现旅游社会总收入13.99亿元，分别是“十五”的19.9倍和21.85倍。商贸流通、住宿餐饮、交通运输等传统服务业明显提升;中介服务、信息传媒等现代服务业迅速发展;银行保险等金融业快速增长，2010年，各项存款余额149亿元，贷款余额84亿元，分别是2005年3.5倍和3倍;城乡市场不断繁荣，社会消费品零售总额达到35亿元，同比增长18.5%，是2005年2.39倍，年均增长19%。

　　三、以建设区域中心城市为目标，城镇化扎实推进

　　城市基础建设步伐明显加快。2010年，市区总人口达到20.88万人，建成区面积达到34.62平方公里，分别比2005年增长33%和9.4%。集中力量实施新区建设大会战，行政办公、学校医院、星级酒店、房产商贸、市政设施等25个项目相继开工，总投资45亿元，年内完成投资16亿元，报业新闻中心建成启用，固原一中、市公安技侦大楼等项目建设进展顺利。道路、供排水、电力通信、供热等路网、管网铺设基本完成，城市框架基本形成，为建设一个现代化新区奠定了坚实基础。集中力量实施城市基础设施攻坚战，开辟了连接新区与老城新通道;新铺设供热管网21公里，新增集中供热面积60万平方米，集中供热普及率达62%以上;新建丝路广场、和谐广场等5处街头景观休闲广场;完成原固原体育场改造和宋家巷民族特色商业居住区改造二期工程。“十一五”期间，市行政中心、固原回中、固原体育馆、宁夏师范学院新校区、市委党校、固原汽车站落户新区;供水厂、污水处理厂、垃圾无害化处理厂、集中供热中心等重点市政工程投入使用。累计新建改扩建市区道路34.5公里，硬化人行道17.2万平方米;建成东海园区、金城花园等一批住宅小区;完成清水河西岸景观建设及河道治理工程。

　　城乡面貌呈现可喜变化。扎实开展“城市管理年”活动，加强违法建设整治，初步建立了市、区、镇、村四级联管机制。实施主要街区亮化美化工程，完成机场路等18条道路路灯安装;建成大原广场、古雁岭生态公园等一批重点城市园林项目，新增城市绿地4818亩，人均公共绿地面积由2005年的7.2平方米增加到10.8平方米，绿化覆盖率达到28.6%;连续3年开展城乡环境综合整治行动，城乡面貌明显改观。

　　县城及中心集镇建设形成新亮点。突出特色，完善功能，在提质扩容上打造了一批新亮点、新景观。西吉县旧城改造和葫芦河县城段治理效果突出，隆德县 “城中村”改造步伐加快，泾源县“一城三区两园”布局初步形成，彭阳县茹河生态园建设和县城绿化美化成效明显，被评为国家园林县城。原州区张易、杨郎小城镇建设走在了全区前列。全市城镇化率预计达到30.85%，比2005年提高13.8个百分点。

　　四、以扩大就业为着力点，劳务产业和全民创业蓬勃发展

　　劳动力转移就业深入开展。2010年，深入开展农民工职业技能“大练兵、大比武”活动，成功举办全市首届农民工职业技能大赛，完成职业技能培训14841人、职业技能鉴定10829人。新建转移就业基地64个，累计达到819个。进一步完善转移就业市场机制，组建劳务集团公司5家、能够组织输出500人以上的劳务中介组织6家。精心组织农村劳动力转移就业“三个万人”输出大行动，全年转移就业30.5万人，实现劳务收入20亿元，转移就业人员人均创收6553元，分别比2005年提高20.8%、156.2%和108.1 %。预计劳务人均纯收入占农民人均纯收入的44.4%。原州区、西吉县、彭阳县被命名为全国农村劳动力转移就业工作示范县(区)。

　　全民创业扎实推进。着力搭建政策、基地、服务、融资、市场、培训“六大平台”，深入开展民营企业家、大学生、返乡农民工、复退军人、下岗失业人员、农村能人“六大创业行动”。2010年，新建创业园区和孵化基地10个，累计达到29个;创办小企业505个，累计达到1448个;培养小老板1105 人，累计达到2136人;创造新岗位5262 个，累计达到13890个;累计开展创业能力培训3946人;成立创业小额贷款担保机构6家，发放担保贷款1.37亿元。

　　城镇就业稳步扩大。认真实施积极的就业政策，采取政府购买公益性岗位、机关事业单位招考、三支一扶、特岗教师、特岗医生招考等措施，2010年新增城镇就业11606人。“十一五”累计实现城镇就业54166人，其中城镇失业人员就业再就业42670人，政府购买公益性岗位安置下岗失业人员2389人，高校毕业生就业9107人。

　　五、以增强发展后劲为动力，生态和基础设施建设成效显著

　　生态建设步伐加快。2010年，完成长城梁生态农业科技示范园二期工程、原州区须弥山河道综合治理、西吉县城北山绿化、隆德县六盘山珍稀植物园、泾源县瓦亭荒山绿化、彭阳县城南北山绿化等6个3000亩以上集中连片绿化点;扎实开展退耕还林补植补造，2002年度 41.8万亩通过国家核查验收;完成水土流失治理面积498平方公里;创建生态示范乡(镇)6个、示范村57个。“十一五”期间，累计完成退耕还林9.5万亩，人工造林105.2万亩，封山育林32.7万亩，道路绿化2132公里，建成贺家湾水源地绿化等一批集中连片绿化点，森林覆盖率达到17.6%。狠抓封山禁牧，培育壮大苗木、林果、枸杞、中药材等后续产业。加大环境保护，四县污水处理厂相继建成，马铃薯淀粉废水治理技术研发成功并推广应用，主要河流水质明显好转。

　　基础设施明显改善。抢抓国家增加投资、扩大内需的政策机遇，重点实施了一批交通、能源、信息、水利等骨干项目。2010年，六盘山机场建成通航，309国道彭阳过境段、马成河至硝口段建成通车，福银高速六盘山镇至沿川子段建设进展顺利，原州区至王洼铁路运煤专线开工，新建农村水泥和沥青公路1120公里;西气东输二线工程固原段建设进展顺利，农村能源建设积极推进;开工建设秦家沟水库、彭堡水源地地下水库。 “十一五”期间，境内高速公路通车里程达到82公里，公路通车里程达到6439公里，新建改建农村公路294条3922公里，实现所有乡(镇)通沥青路，72.7%行政村道路硬化，立体交通网络初步形成;清水河330千伏变电站建成投入使用，率先在西北地区实现了农村户户通电;全市所有行政村建成了农村信息服务站，每百人拥有固定电话15部、移动电话45部，覆盖城乡的信息网络体系逐步形成。兴修旱作基本农田65.3万亩，新增节水灌溉面积12.6万亩;完成东山坡引水、原州区东部安全饮水、固西引水等一批重大水利设施建设项目和62座病险水库除险加固、50处农村安全饮水工程，抗旱减灾能力进一步提高。{CACOPAGE}

　　六、以健全社会保障体系为重点，民计民生切实改善

　　社会保障体系不断完善。率先在全区建立市区失地农民最低生活保障制度;实现了职工医疗、生育保险市级统筹，扎实推进统筹城乡居民医疗保险试点，全面启动实施了新农保试点;积极解决社会基本养老保险、医疗保险历史遗留问题。不断完善社会救助机制，全市纳入城乡低保18.6万人，实施医疗救助27.34万人次，为城乡10515名80岁以上生活困难老人发放了高龄津贴，为全市69301户农村低保户和18351户城市低保户分别发放30元和50元“两节”慰问金。以基本养老、医疗、失业、工伤和生育保险为主要内容的社会保障体系基本建立。

　　人民生活水平不断提高。城乡居民衣食住行条件大为改善。“米袋子、菜篮子”进一步丰富，稳定解决了群众温饱问题;群众收入普遍提高，购买能力显著增强。住房公积金制度覆盖面进一步扩大，归集总额达到12.01亿元，贷款总额达到6.88亿元;全市累计完成廉租住房等保障性住房53.26万平方米，实施危房危窑改造5.7万户。认真实施“家电下乡”等惠民政策，购车、购房和旅游等成为消费热点;城乡居民各项储蓄存款余额达74亿元。

　　扶贫开发稳步推进。加强闽宁对口扶贫协作，扎实做好定点帮扶、整村推进和社会扶贫工作。2010年，共整合各类资金4.08亿元，完成第三批164个整村推进扶贫开发年度任务。争取互助资金3345万元，极大改善了149个贫困村的生产生活条件，全市贫困人口减少5.1万人。“十一五”期间，累计投入各类扶贫资金11.53亿元，贫困人口减少到45万人。

　　七、以构建和谐社会为根本，社会事业协调发展

　　教育科技人才事业加快发展。深入开展教育强县(区)创建和基本普及高中阶段教育工作，原州区、隆德县、彭阳县通过自治区教育强县(区)验收。认真落实“三免一补”、困难学生资助、营养早餐等教育普惠政策。编制完成《固原市地方课程教材》初稿。“十一五”期间，全市实现“两基”教育目标，学前教育、职业教育、民族教育、特殊教育、成人教育快速发展。大力实施教育质量提升工程、中小学教师和校长能力建设计划，教育教学质量明显提高。大力实施中小学校舍安全改造工程，校舍面积达到162万平方米，比2005年增加44万平方米，办学条件明显改善，有效缓解了“大班额、大通铺”问题。全市适龄儿童入学率、初中阶段毛入学率、高中阶段毛升学率分别达到99.7%、106.4%和85.1%，比2005年分别提高0.95个、20.1个和35个百分点。大力推进科技创新，科技特派员创业行动成效突出，建成科技示范园区6个。科技对国民经济增长的贡献率达到39%，比2005年提高2个百分点。编制完成了《固原市中长期人才发展规划纲要(2010—2020年)》，完善了人才引进、选拔、培训、教育机制。

　　文化事业大发展大繁荣。实施精品文化工程和县(区)特色文化工程，举办了六盘山精神暨西海固文学艺术研讨会，出版发行了《六盘山文化丛书》、《六盘山民间故事》等刊物，编排了《王洛宾的花儿情》等优秀剧目。组织了 “花儿漫六盘”电视大奖赛等文艺展演，非物质文化遗产和文物保护成效显著。成功举办2010年全国群众健身大会暨首届六盘山登山节，全民健身活动蓬勃开展。加快文化惠民工程建设，广播电视覆盖率达到98%，农村8套以上广播电视节目入户率达到93.3%。“十一五”期间，西吉县、隆德县被评为“全国文化先进县”，原州区、彭阳县被评为“全国文物保护先进县”。

　　医药卫生工作不断加强。加快医药卫生体制改革，推进人人享有基本医疗卫生服务试点工作，认真落实药品“三统一”政策，提前实现国家基本药物制度全覆盖。市人民医院门诊住院综合楼、市妇幼保健院迁建、隆德县医院迁建、彭阳县中医院住院部建成使用，市中医院、西吉县医院迁建工程进展顺利。“十一五”期间，对所有乡镇卫生院实施了改扩建，实现了村村都有合格卫生室目标，医疗卫生服务体系逐步完善，住院床位达到3390张，比2005年增加1156张。城镇居民医疗保险、新农合参合率分别达到95.2%和93.3%，群众“看病难、看病贵”问题得到有效缓解。

　　人口和计划生育工作取得新成效。严格责任落实，加快实施“少生快富”、信息化建设、“星级乡镇”创建工程，完成五县(区)县级计生服务站改扩建和85%的乡(镇)计生服务站标准化建设。实施“少生快富”工程5031例，累计实施29551例，创建“少生快富”示范户584户。全市人口出生率控制在17.24‰，比2005年下降2.18个千分点，出生政策符合率达到83.2%，比2005年提高6.07个百分点。组织开展了全国第六次人口普查工作。

　　和谐共建工作扎实推进。继续开展矛盾纠纷排查调处和领导干部“大接访”活动，有效化解了一批矛盾纠纷和信访积案。加强社会治安综合治理，深入推进新一轮“平安固原”建设，严厉打击各类犯罪，营造良好的社会环境。认真贯彻党的民族宗教政策，扎实开展民族团结进步、和谐宗教活动场所创建活动，依法加强宗教事务管理，妥善处理宗教内部矛盾，保持宗教领域和谐稳定。严格落实安全生产责任制，集中开展各类安全隐患排查整治，安全生产形势总体平稳。加强食品药品安全监管力度，维护群众切身利益。进一步完善应急管理制度，开展了地震、消防等应急救援演练，应对突发公共事件能力不断提高。积极开展精神文明创建活动，基层民主建设得到巩固，国防动员和双拥共建工作不断加强。外事侨务、人防、统计、粮食、供销、气象、档案、地方志、老龄、残疾人、妇女儿童等各项工作都取得新进展。

　　八、以提高执行力为突破口，政府自身建设不断加强

　　坚持把“学习、实干、突破”作为主题，把提高执行力、奋力抓落实作为提升政府效能、落实目标任务、解决突出问题的重要举措，贯穿于政府工作的全过程。通过深入开展学习实践科学发展观、先进性教育、实施西部大开发战略等一系列学习活动，进一步解放思想，明晰发展思路，推进科学发展。全面推进依法治市进程，“五五”普法任务圆满完成。坚持依法行政，自觉接受人大依法监督、政协民主监督、群众评议监督和舆论监督，五年累计办理人大代表议案建议132件、政协委员提案建议745件，办复率100%。加快电子政务建设，建立健全了市、县、乡(镇)、村四级便民服务网络，积极推行政务公开，政府议事规则、民主决策、信息公开等制度进一步完善，群众关注的热点难点问题得到及时妥善处理。狠抓机关作风建设，办事质量和行政效能进一步提高。创新干部管理机制，实行目标管理考核，激发了各级组织和广大干部的干事激情和创新活力。严格落实党风廉政建设责任制，全面推行廉政风险防范管理和廉政勤政承诺制，加强重点行业、重点领域、重大项目资金管理使用的审计监督和行政监察，政府系统反腐倡廉工作全面推进。

　　各位代表，2010年各项目标任务的胜利完成，标志着“十一五”期间我市经济社会发展实现了具有重要意义的突破和跨越。这五年，是我们认真践行科学发展观，深化市情认识，进一步明确发展思路，转变经济发展方式，经济结构不断优化的五年;这五年，是我们抢抓发展机遇，积极应对各种挑战，经济增长逐年加快，活力增强，成效显著的五年;这五年，是我们实施城市带动战略，加快推进宁南区域中心城市建设，促进城乡统筹发展，城乡面貌变化最大的五年;这五年，是我们谋长远打基础，千方百计争项目，不断改善发展环境，增强发展后劲的五年;这五年，是我们认真落实国家、自治区一系列惠民政策，集中力量办大事，尽心竭力办实事，社会事业发展进步最快、人民群众得实惠最多的五年;这五年，是我们正确处理改革发展稳定的关系，加强民族团结，维护社会稳定，凝心聚力，团结拼搏，和谐社会建设取得新进步的五年。

　　各位代表，“十一五”的发展，为我市全面建设小康社会、实现科学发展、跨越发展奠定了坚实基础。这些成绩的取得，是自治区党委、政府和市委坚强领导的结果，是市人大、市政协有效监督和社会各界大力支持的结果，是全市广大干部群众团结奋斗、顽强拼搏的结果。在此，我代表市人民政府，向支持政府工作的各位代表和委员，向辛勤工作在全市各条战线上的全体劳动者，向各民主党派、工商联、人民团体和社会各界人士，向中央、自治区驻固各单位、人民解放军、武警官兵和公安干警，向所有关心固原建设和发展的朋友们，表示崇高的敬意和衷心的感谢!

　　在肯定成绩的同时，必须清醒地认识到，我们的工作与人民群众的期望还有一定差距，经济社会发展中还存在一些突出矛盾和问题。主要是：自然条件差，生态环境脆弱，人口与资源、环境承载能力矛盾突出;经济总量小，结构不合理，发展方式粗放，自我发展能力弱，市场化程度低，与全区其他市的发展差距拉大;对外开放水平不高，招商引资难度大，发展环境有待进一步优化;基础设施建设滞后，基本公共服务能力不强，保障和改善民生任务艰巨;贫困面广程度深，扶贫攻坚的任务十分繁重;干部群众的发展能力和信心还需提高增强。这些矛盾和问题，我们将在今后的工作中认真加以解决。{CACOPAGE}

　　“十二五”时期发展战略任务

　　“十二五”时期，是我市深入实施新一轮西部大开发战略、推进经济发展战略转型的重要时期，也是全面建设小康社会承前启后的关键阶段。我们必须树立强烈的机遇意识、忧患意识、责任意识和追赶意识，奋力拼搏抓落实，开拓创新求突破，推动经济社会各项事业科学发展、跨越发展。

　　“十二五”时期经济社会发展的指导思想是：高举中国特色社会主义伟大旗帜，以邓小平理论和“三个代表”重要思想为指导，深入贯彻落实科学发展观，顺应全市人民过上更好生活新期待，深入实施西部大开发战略，认真落实自治区党委、政府《关于促进固原经济社会加快发展的若干意见》，大力弘扬“不到长城非好汉”的革命精神，以科学发展为主题，以经济发展战略转型为主线，全力实施“一五五”工程，奋力推进新型工业化、城镇化、农业产业化，奋力推进以生态移民为重点的扶贫开发，奋力推进基础设施和生态环境建设，更加注重改革开放，更加注重优化发展环境，更加注重项目建设，更加注重保障和改善民生，更加注重民族团结和社会稳定，努力走科学发展、跨越发展之路，为与全国、全区同步迈入小康社会奠定坚实基础。

　　“十二五”时期经济社会发展的主要奋斗目标是：地区生产总值力争比2010年翻一番，年均增长13.5%左右;地方财政一般预算收入年均增长20%以上;全社会固定资产投资年均增长25%以上;城镇居民人均可支配收入年均增长11%;农民人均纯收入力争翻一番，年均增长12%。人口自然增长率控制在11‰以内，城镇登记失业率控制在4.4%以内，完成自治区下达的节能减排任务。

　　要实现以上目标任务，必须着力抓好五个方面的重点工作。

　　一、着力推动经济发展战略转型，形成经济发展新格局

　　坚持一二三产业协调发展，做优一产，做强二产，做活三产，使三次产业比例由2010年的27.8：24.9：47.3调整到2015年的19：38：43，初步形成“工业主导、多业并举”的经济发展新格局。

　　大力提升工业水平。坚定不移地实施兴工强市战略，培育产值过100亿元的盐化工及建材业、过50亿元的煤电油产业和过30亿元的特色农副产品加工业等三大产业集群，力争工业增加值年均增长29%。加快推进园区建设。按照产品项目、公用辅助、物流传输、环境保护、管理服务“五个一体化”模式，把固原盐化工循环经济扶贫示范区建成自治区级循环经济示范区，重点开发以烧碱、纯碱为主的盐化工产品，到2015年盐化工示范区对全市工业经济的贡献率达到50%以上。依托石灰岩资源和粉煤灰等工业废料，发展干法水泥、空心烧结砖等新型建材业。完善更加优惠的政策、措施，加大招商引资力度，支持固原经济开发区、宁夏(西吉)闽宁合作产业园等园区建设。加快培育煤电油产业。建成王洼千万吨级大型煤炭生产基地，争取开工六盘山热电厂2×670兆瓦扩建和盐化工示范区2×350兆瓦自备电厂建设，到2015年煤炭生产能力突破1000万吨，煤电装机容量达到252万千瓦以上。加快油气资源勘探开采进度，推进石油就地转化增值。加快建设和引进风力发电、光伏产业项目。争取西气东输二线天然气接通到各县城。加快改造传统产业。提升特色农副产品、中药材等加工水平，扶持壮大10家龙头企业，大力实施技术改造和品牌战略，提升市场竞争力。

　　大力发展生态农业。坚决落实国家、自治区强农惠农政策，夯实农业基础，加快优势特色产业发展。力争农业增加值年均增长6%。坚持最严格的耕地保护制度，稳定粮食生产。根据地域特点和发展优势，规划在黄土丘陵沟壑区建设以草畜、马铃薯、小杂粮为主的农牧复合产业带，在河谷川道区建设以设施农业为主的现代农业产业带，在土石质山区建设以养牛、育苗、中药材为主的特色农业产业带。精心打造23个自治区级、27个市级生态农业示范基地，辐射带动农业特色优势产业扩量提质增效。马铃薯种植面积稳定在200万亩以上，建成三级脱毒种薯繁育推广体系，原原种生产能力达到2亿粒，打造全国种薯基地;肉牛饲养量达到100万头以上;设施农业稳定在27万亩以上。按照“一县一品”的要求，建设以原州区为中心的生态型冷凉蔬菜和枸杞生产基地、以西吉为中心的马铃薯和西芹生产基地、以隆德为中心的中药材和瘦肉型猪生产基地、以泾源为中心的肉牛和苗木生产基地、以彭阳为中心的辣椒和经果林生产基地。积极引导、规范土地流转方式，提升农业产业化水平，打造“六盘山”绿色农产品品牌，扶持培育10个产值上亿元的农业产业化龙头企业，形成集群优势，打造全国生态农业示范区。

　　大力培育第三产业。依托区位优势，加快发展服务业。力争服务业增加值年均增长11.3%。按照“立足宁南、辐射周边、对接三省”的思路，建设固原火车站农产品货运物流、三营火车站工业品物流、清水河综合物流等园区，培育年销售收入上亿元的物流企业10家，建设马铃薯、蔬菜、建材等自治区级批发市场和优势特色农产品交易市场，打造西兰银交汇中心物流集散基地。立足旅游资源优势，建设六盘山、须弥山5A级景区，加快建设六盘山红色旅游、生态旅游及文化休闲避暑度假基地。改造提升商贸餐饮、交通运输等传统服务业，大力发展文化、金融、保险、信息等现代服务业，加快传统服务业向现代服务业转变。

　　二、着力推进城镇化进程，打造宁南区域中心城市新形象

　　坚持科学定位、统筹规划、完善功能、产业支撑、梯次推进，加快宁南区域中心城市、特色县城、中心集镇三级城镇体系建设，城镇化率达到40%以上。

　　加快宁南区域中心城市建设。围绕建设“生态文化山城”，修编固原城市总体规划，市区建成区面积达到37平方公里。优化城市功能布局，加快建设集工业、商贸、物流、文化旅游为一体的宁南区域中心城市，增强城市的辐射带动能力。加快集行政办公、文化教育、商务居住等为一体的新区建设;加快老城基础设施配套改造，建设功能完善、管理有序、环境优美、生活便利的商贸居住休闲区;优化固原经济开发区功能布局，建设支撑城市发展的产业区。完成101省道固原市区段改线等市区道路工程，构建四通八达的城市公路网。完善城市管理体制，加强城市经营和管理，争创自治区级文明城市、卫生城市、园林城市。

　　加快特色县城建设。根据各县产业发展方向和民俗文化特征，编制和完善县城建设控制性详细规划，建设西吉风情县城、隆德文化县城、泾源旅游县城、彭阳生态县城，使之成为承接区域中心城市产业转移、辐射和带动小城镇发展的重要引擎。

　　加快中心集镇和新农村建设。整合项目和资源，完成三营镇、兴隆镇、沙塘镇、泾河源镇、古城镇等16个中心集镇建设，提高城镇综合承载能力，形成特色鲜明、基础完善、功能互补的小城镇发展格局。做好社会主义新农村规划建设，实施好水、电、路、气、房和环境“六到农家”工程，努力建设一批具有一定辐射带动能力的新农村示范村。

　　三、着力实施生态移民工程，开辟扶贫开发新途径

　　抢抓国家推进秦巴—六盘山集中连片特殊困难地区扶贫开发新机遇，强力实施生态移民、劳务移民和产业移民，促进固原以农业为主导的扶贫开发向工农并重多产业协调发展转变。完成生态移民23.25万人，减少贫困人口26万人。

　　全力推进生态移民工程。把生态移民作为从根本上解决贫困问题、同步进入小康的重大举措，举全市之力打一场攻坚战。采取县外与县内移民相结合的方式，用5年时间，将干旱片带、环境恶劣、生态脆弱、不宜人居地方的贫困人口搬迁出来。配套完善移民新区公共服务设施，加强移民实用技术和创业能力培训，大力发展致富产业，增强移民自我发展能力，从根本上解决农村贫困人口的生产和发展问题。

　　全力推进劳动力转移工程。继续推进劳动力大市向劳动力强市、打工型向技能创业型转变，大力开展大规模的技能培训和“大练兵、大比武”活动，提升务工人员技能素质。培育壮大专业化劳务集团公司，大力发展劳务中介服务组织、劳务经纪人队伍。提升电焊电工、电子装配、缝纫制鞋、家政保安、建筑装潢“六大品牌”。巩固扩大福建、新疆和内蒙古、京津唐、长三角和珠三角、宁东及沿黄城市带、中东涉外“六大板块劳务输出基地”。新建劳务基地250个以上，年转移就业30万人以上，创收25亿元以上，人均年创劳务收入8000元以上。

　　全力推进扶贫开发工作。认真实施“六盘山区集中连片特殊困难地区扶贫开发攻坚计划”，扎实做好新一轮整村推进扶贫开发工作，加大产业扶贫、移民扶贫、科技扶贫、项目扶贫力度，扩大互助资金扶持面，增强贫困人口自我发展能力。进一步加强社会帮扶力度，拓宽定点帮扶工作领域，提升闽宁对口帮扶协作水平，构建“大扶贫、大开发”新格局。

　　四、着力加强基础设施和生态建设，增强发展新活力

　　将扩大投资作为“十二五”及今后一个时期经济增长的主要拉动力，进一步加快基础设施建设，夯实发展基础，进一步强化生态建设与保护，走可持续发展之路。

　　加快基础设施建设。坚持山、水、田、林、路综合治理，兴修旱作基本农田50万亩，实现农业人口人均3亩高标准基本农田目标。建成福银高速六盘山镇至沿川子段、兰青高速毛家沟至东山坡段和原州区至王洼铁路运煤专线，实现所有行政村通沥青水泥路目标。力促开工建设宝中铁路复线、固原至定西铁路和鸳鸯湖至彭阳、固原至西吉高速公路，完善立体交通网络。

　　加快水资源综合开发利用。推进“北扬黄河水、南引泾河水、就近利用库井水”配制工程体系建设，完成宁夏(中南部)固原地区城乡饮水安全水源工程，年引水3980万立方米;确保固海扬黄扩灌工程年扬水4000万立方米;新建9座中小型水库，新增库容3000万立方米，完成37座病险水库除险加固任务，建成农村人饮工程49处。在解决城乡居民安全饮水的基础上，统筹解决生态用水、农业用水和工业用水问题。加强水源地保护，严格水资源管理，全面推进节水型社会建设。

　　加快构筑西部生态安全屏障步伐。根据地形地貌不同类型，在土石质山区建设以大六盘生态经济圈为核心的水源涵养林生态功能区，新造水源涵养林60万亩，完成天然林保护工程封育40万亩;在黄土丘陵沟壑区建设以治理水土流失为主、乔灌草结合的生态林牧复合功能区，争取15度以上186万亩坡耕地实施退耕还林;在河谷川道区建设以高标准农田防护林网和特色经济林为重点的林业产业示范区;在城镇重点构建城市森林生态体系，村镇绿化网络体系，完成城乡大环境绿化10万亩、绿色通道1000公里、乡村四旁植树2000万株。到2015年，全市森林覆盖率达到25%，治理水土流失面积1000平方公里，治理程度达到85%以上，争做宁夏构建祖国西部生态屏障的先行区。

　　加强环境保护工作。坚持预防为主、综合治理原则，强化源头污染防治和生态保护，大力推进循环经济和低碳技术的研发运用，支持能源化工企业节能减排。强化主要河流和重点污染源监管，加强水污染防治工作，加大淀粉废水治理实用技术运用。加强农村环境保护，开展农村环保小康行动，提高城乡环境质量。{CACOPAGE}

　　五、着力保障和改善民生，构建和谐新固原

　　坚持实施民生计划，推进基本公共服务均等化，加强社会建设，构建广大人民群众在共建中共享、共享中共建的和谐社会。

　　进一步扩大创业就业。继续实施全民创业工程，新建创业园区和孵化基地12个，新培育小企业(小老板)1500个，新增就业岗位1.5万个，新增城镇就业3万人。积极开发公益性就业岗位，支持和鼓励劳动者自主创业和自谋职业，解决好大学生、退役军人和困难家庭的就业问题。

　　进一步完善社会保障体系。做好社会“五险”扩面提标，增强社会保障能力。加快统筹城乡居民医疗保险，参保率达95%以上。全面完成新农保试点工作。认真落实市区失地农民最低生活保障制度。健全覆盖城乡的社会救助体系，进一步提高社会救助标准。不断扩大住房公积金制度覆盖面，加快保障性安居工程建设，新建廉租房、公共租赁房等保障性住房4.7万套，改造农村危房危窑4万户。大力发展残疾人事业、老龄事业和慈善事业。

　　进一步发展教科文卫等社会事业。全面贯彻落实《国家中长期教育改革和发展规划纲要》，深化教育教学改革，优化教育资源配置，促进城乡教育均衡发展，切实加强教师队伍建设，提高教育教学质量;完成全市所有中小学校舍安全改造工程。围绕优势特色产业加强科技攻关，科技对国民经济增长的贡献率达到45%。繁荣文化事业、发展文化产业，深入开展群众性文化体育活动和全民健身活动，继续办好六盘山系列节事等活动;新建市广电中心和4个县级图书馆等文化项目。深化医药卫生体制改革，完善农村三级卫生服务网络和城市社区卫生服务体系，创建人人享有基本医疗卫生服务示范市;新建市医院内科大楼、市县(区)卫生监督所和19个社区卫生服务中心(站)。深入实施《固原市中长期人才发展规划纲要(2010—2020年)》，以专业技术人才和农村实用人才为重点，统筹抓好各类人才队伍建设。完善人口和计划生育利益导向机制和奖励扶助政策，深入实施“少生快富”工程、优生促进工程和出生缺陷干预工程，严格控制人口数量，不断提高人口素质，人口出生率控制在16.5‰以内。

　　进一步加强社会管理。推进法治政府建设，深入实施“六五”普法宣传教育工作;深入推进以“社会矛盾化解、社会管理创新、公正廉洁执法”三项重点工作为载体的“平安固原”建设，建立健全群体性事件应急处置机制，依法严厉打击各种犯罪活动。加强和改进信访工作，继续开展矛盾纠纷排查化解、信访积案化解、领导干部大接访等活动，解决群众的合理诉求。全面贯彻党的民族宗教政策，深入开展民族团结进步创建活动。严格落实安全生产责任制，最大限度地减少安全事故的发生。进一步完善应急管理机制，提高应对突发公共事件的能力。深化体制改革，扩大对外开放，优化发展环境，建设与市场经济相适应的公共服务型政府。

　　2011年的主要工作目标和任务

　　2011年是“十二五”规划开局之年，也是推进经济发展战略转型的关键一年，做好今年工作，意义重大。全市经济社会发展的主要预期目标是：地区生产总值增长13.5%，地方财政一般预算收入增长20%，全社会固定资产投资增长26%，社会消费品零售总额增长15%，城镇居民人均可支配收入增长11%，农民人均纯收入增长12%。人口自然增长率控制在11.8‰以内，城镇登记失业率控制在4.4%以内，单位GDP能耗、化学需氧量和二氧化硫排放量均控制在自治区下达的目标以内。

　　为实现以上目标任务，我们将着力抓好事关全市经济社会发展的14件大事和改善民生的28件实事，重点做好以下八个方面的工作。

　　一、加快建设盐化工循环经济扶贫示范区，提升工业化水平

　　以岩盐、煤炭和新能源开发为重点，以招商引资和大力发展非公有制经济为抓手，加快推进工业主导型经济发展，工业增加值增长27%。

　　盐化工循环经济扶贫示范区建设要实现重大突破。完善盐化工示范区总体规划和基础设施详细规划，全面完成示范区“二纵二横”主干道及绿化、供排水、信息网络和供电网络等基础设施建设。开工建设采输卤、真空制盐、PVC等项目，做好示范区2×350兆瓦热电厂前期工作。积极探索市、县(区)共建示范区新机制，除农产品加工企业外，一般竞争性工业企业全部集中到示范区。鼓励民营资本参与盐化工产业下游产品的开发和加工。加快固原经济开发区建设，做好产业规划布局，着力发展纺织等轻工产业。

　　煤电建材产业要实现新发展。确保六盘山热电厂正常运行和王洼二矿年产150万吨原煤生产线全面投产，完成银洞沟煤矿年产300万吨原煤生产线主体工程，力争西吉月亮山风电厂一期49.5兆瓦项目建成并网发电，加快六盘山热电厂2×670兆瓦扩建项目前期工作。加快推进彭阳油气资源勘探开发利用。积极引进光伏产业，支持日产2500吨新型干法水泥旋窑生产线等重点项目建设，支持利用粉煤灰等工业废料发展新型建材业。

　　农副产品加工业要实现新提升。支持马铃薯淀粉加工企业发展淀粉精深加工，完成荣发草业年产5万吨饲料颗粒生产线、隆昊公司年产2万吨肉类产品生产线二期、德邦生物年产1万吨菊芋粉加工、明德中药年产5000吨中药饮片加工等项目建设，促进宁夏六盘山药业公司同伊正医药公司合并重组。

　　节能减排要实现新目标。强化目标责任，狠抓重点耗能企业的节能降耗工作，加快推进马铃薯淀粉废水污染治理，确保污水处理厂正常运行，完成自治区下达的污染物总量减排任务。继续抓好公共节能，树立全社会节能意识。

　　招商引资要实现新跨越。深入开展“争项目和招商引资推进年”活动，主动积极对接东部产业转移，完善招商机制，创新招商方式，拓宽招商领域，引进实际到位资金20亿元以上。依托中阿经贸论坛、宁洽会等会展活动，大力开拓中东、马来西亚等国际市场，完成外贸出口额200万美元以上。

　　二、加快特色优势产业发展，提升生态农业质量和效益

　　紧紧围绕农民增收，加快调整农业产业结构，培育特色优势产业，促进农业扩规提质增效。农业增加值增长6.1%，粮食总产量稳定在60万吨以上。

　　在培育特色优势产业上求突破。认真实施农民收入倍增计划，加快建设六盘山生态农业示范基地。实施特色优势产业提升工程，新建存栏500头以上肉牛示范村30个，存栏200头以上肉牛养殖园(区)10个，肉牛饲养量达到82万头;马铃薯种植面积稳定在200万亩以上，切实加快三级脱毒种薯繁育推广体系建设，生产原原种1亿粒，种薯扩繁21万亩，建设相对集中连片的万亩种薯基地5个;积极引进大企业、大公司促进设施农业大发展、快发展，新建设施农业5万亩，完成以覆膜保墒为主的旱作节水农业94万亩;扩大中药材、冷凉蔬菜、苗木、林果、枸杞、花卉、菊芋、小杂粮等区域特色种植。

　　在提升农业产业化经营上求突破。建立产业化龙头企业扶持激励机制，各县(区)新引进和发展1家投资5000万元以上的农业产业化龙头企业。积极兴办科技、信息、农资、农产品购销服务等各类专业合作经济组织，每个县(区)至少发展5家。实施品牌战略，培育“六盘山”农产品品牌，提高市场竞争力。健全农产品质量安全体系，做好动植物疫病防控。

　　在劳动力转移就业上求突破。实施劳动力转移就业工程，实现农村劳动力转移就业30万人以上，劳务总收入达到20亿元以上。深入开展技能培训和“大练兵、大比武”活动，完成职业技能培训1.3万人、职业技能鉴定1万人;大力发展劳务中介组织、劳务经纪人队伍，进一步完善增强专业化劳务集团公司的服务带动能力，继续巩固和拓展劳务基地，新建劳务基地50个;加强劳务输出机构管理，切实做好务工人员维权工作。

　　在生态移民和扶贫开发上求突破。奋力实施生态移民工程，组织发动，协调配合，服务督促，全面完成市外移民2.99万人;科学制定市区、县城、中心镇移民规划，配套完善公共服务设施，完成市内移民1.66万人;推进劳务移民、教育移民、产业移民，确保“搬得出，稳得住，能致富”。坚持开发式扶贫，千方百计落实各类扶贫资金，不断扩大互助资金覆盖面;拓展社会扶贫领域，扎实做好闽宁对口帮扶工作，提升扶贫开发整体水平。{CACOPAGE}

　　三、加快宁南区域中心城市建设，提升城镇带动辐射能力

　　坚持规划与建管并重，突出自然特色、地域特点和文化品味，加大市区建设和改造力度，扩大县城和中心镇规模，加快推进三级城镇体系建设，全市城镇化率达到32%。

　　扎实推进宁南区域中心城市建设。加快市区建设步伐，实施和谐路、体育路等8条道路和管网建设，加快华祺、民航等大酒店和盛世华都、御景天城等一批住宅小区的建设。加快旧城改造，实施宋家巷民族特色商业居住区三期工程，完成固胡路等5条城市道路改造，硬化一批城市街巷道，争取立项建设固原第二污水处理厂;完成市区集中供热管网改造和天然气管网建设。实施以廉租住房、公共租赁房等为主的保障性住房1.086万套。继续狠抓房地产市场和建筑市场的整顿规范。完善城市公用设施，增强城市服务功能，提升城市品位和形象。

　　扎实推进县城和中心集镇建设。按照各县县城特色定位，大力实施一批城市公共服务建设项目。抢抓国家实施百县中心镇建设工程机遇，加快中心集镇规划和基础设施建设，每县(区)建设1-2个特色鲜明的中心集镇。将新农村建设与推进城镇化有机结合起来，完善村庄规划，建设布局合理、资源共享、各具特色的社区化新农村示范村20个，完成农村危房危窑改造8000户。

　　扎实推进城市管理。深入开展“城市建设与管理提升年”活动，完善市、区、镇、村四级分工负责、各司其职、共建共管的城市管理长效机制，切实治理违法建筑。探索城市公共服务设施社会化管理新途径，加大社区服务的基础性工作，加强街道、公园、广场、绿地等公共服务设施管护。全面实施环境综合整治，建立市容市貌“严管街”和门前三包“样板街”，重点整治城乡结合部、背街小巷等区域脏、乱、差现象，启动自治区级园林城市创建工作。

　　四、加快发展旅游商贸物流业，提升第三产业发展层次

　　依托区域中心城市和旅游资源优势，大力发展以旅游、物流、商贸为重点的第三产业，着力培育新的经济增长点。三产增加值增长11%。

　　大力发展旅游业。围绕六盘山红色旅游和生态旅游及文化休闲避暑度假基地建设，组建六盘山旅游集团公司，推进旅游资源整合开发。实施六盘山国家森林公园、老龙潭、须弥山、火石寨、白云寺等重点景区基础设施建设工程。开工建设须弥山景云寺，力争启动国家红色旅游二期建设项目。加强与周边省市的旅游合作和资源共同开发，办好“六盘山山花旅游节”、“清凉六盘·消夏之旅”等节事活动。全年接待游客170万人次以上，实现旅游社会总收入4.5亿元以上。

　　加快发展商贸物流业。全力推进尚都国际商贸中心、派胜世茂城、鑫祥商业区等建设步伐，完成瑞丰一级综合农产品批发市场、彭阳县综合批发市场建设和西吉县果蔬批发市场改扩建项目;继续实施 “万村千乡”市场工程、“三新工程”和 “家电下乡”惠民工程。围绕西兰银交汇中心物流集散基地建设，启动建设清水河物流产业带(园)工程，力争引进1-2家投资5000万元以上的物流企业，培育年销售收入上亿元的流通企业2家。

　　积极发展现代服务业。大力发展连锁经营、物流配送等现代服务业。培育物业管理、中介服务、信息咨询、商务会展、家政服务等新兴服务业。大力发展金融保险业，建立健全银政企定期沟通机制，增强信贷融资能力和金融服务水平。

　　五、加快基础设施和生态环境建设，提升可持续发展能力

　　抢抓国家实施西部大开发等战略机遇，坚持把项目作为发展的重要载体，谋划、争取、实施、储备一批打基础、增后劲、促转型的重大项目。

　　突出基础项目建设。完成福银高速六盘山镇至沿川子段、兰青高速毛家沟至东山坡段、101省道固原市区段改线、原州区至王洼铁路运煤专线年度建设任务;全力做好宝中铁路复线和固原火车站、三营火车站改扩建项目的前期工作;全面完成1000公里农村公路建设任务。加快水利设施建设步伐，实施宁夏(中南部)固原地区城乡饮水安全水源工程和清水河马饮河段河道治理工程，加快秦家沟水库、彭堡水源地地下水库等重点项目建设，争取开工建设吴庄等15座小型病险水库除险加固工程，开工建设13处农村饮水安全工程，解决12万农村人口饮水困难。

　　狠抓生态项目建设。围绕“三河源”水源保护和大六盘生态经济圈建设，认真实施 “六大绿化工程”，完成人工造林20万亩、城乡大环境绿化2万亩、道路绿化200公里，完成退耕还林补植补造80万亩，创建生态示范乡(镇)5个、生态示范村50个。实施长城梁生态农业科技示范园三期工程，各县(区)新建1处3000亩以上的集中连片绿化点。治理水土流失面积200平方公里，每县(区)集中打造1个5000亩以上的农田水利基本建设综合治理示范点。同时，要严格项目管理，落实责任措施，确保项目质量，为加快发展提供有力支撑。

　　六、加快发展社会事业，提升市民综合素质

　　坚持统筹兼顾，加快教育、科技、卫生、文化等社会事业发展，促进经济增长与社会发展相协调。

　　优先发展教育事业。巩固提高义务教育普及成果，西吉、泾源两县完成教育强县创建任务，实现教育强市创建目标。原州区、隆德县、彭阳县基本普及高中阶段教育工作通过自治区评估验收。加强教师队伍建设，统筹培训城乡教师。实施好中小学校舍安全改造工程和第二期“百标工程”，完成固原一中迁建，开工建设西吉五中、隆德高级中学和原州区第七中学、第十三小学、第十六小学、第四幼儿园、第六幼儿园等重点项目。大力发展学前幼儿教育、特殊教育和职业教育，做好固原民族职业技术学院申报高等职业(专科)学院各项准备工作，加强东西联合办学，提高职业教育入学率和就业率。

　　加快科技创新步伐。围绕优势特色产业发展，开发引进推广一批新技术、新工艺、新产品，推进企业技术改造和产业升级。深入开展科技特派员创业行动，加快科技园区建设，促进科技成果转化。加强科普教育，提高全民科技素质。

　　推进医药卫生体制改革。围绕“人人享有基本医疗卫生服务示范市” 创建目标，健全农村三级卫生服务网络和城市社区卫生服务体系，认真落实国家基本药物制度和自治区药品“三统一”政策。完成市中医院、西吉县医院迁建工程，开工建设市精神卫生中心。加强食品药品安全监管。做好疾病预防控制、妇幼保健、中医药、爱国卫生等工作。

　　大力发展文化事业。发挥“红色六盘、萧关古道、丝路古城、花儿家乡”文化资源优势，举办中国西部“花儿”歌会暨第三届“花儿漫六盘”大奖赛等活动，组织开展文化下乡、广场文化、社区文化展演活动。大力发展公共体育事业，倡导全民健身。继续实施农村广播电视村村通、农村数字电影放映和农家书屋等文化惠民工程。加强文化市场管理和非物质文化遗产及文物保护工作，做好第三阶段文物普查工作。开工建设市广电中心。实施文明创建工程，广泛开展文明市民、文明单位、文明乡(镇、社区)等精神文明创建活动。

　　加强人口与计划生育工作。严格目标责任管理，扎实推进 “少生快富”工程，加大流动人口管理力度，积极开展“优质服务先进县” 、“星级乡镇”和“少生快富”示范户创建活动，全市人口出生率控制在17.71‰以内，出生政策符合率达到83.2%以上。

　　七、加快构建社会保障体系，提升城乡居民生活质量

　　坚持把保障和改善民生作为加快转变经济发展方式的根本出发点和落脚点，建立健全基本公共服务体系，让人民群众得到更多实惠。

　　大力推进全民创业。启动全国创业型城市建设工作，在巩固原有创业基地基础上，每个县(区)再培育1—2个有特色的创业园区或孵化基地;继续开展 “六大创业行动”，新创办小企业300个以上，培养小老板300人以上，创造新岗位3000个以上;继续采取政府购买公益性岗位等措施，帮助更多的残疾人、困难群体实现就业，全年实现城镇新增就业6000人。

　　加快完善社会保障体系。全面推行新型农村养老保险试点工作和城乡居民医疗保险市级统筹，参保率分别达到80%和93%以上。进一步扩大养老、失业、医疗、工伤、生育保险和住房公积金制度覆盖面，完善市区失地农民最低生活保障制度，加快建立覆盖城乡的社会保障体系。进一步做好社会救助工作，深入推进城乡低保规范化管理，做到应保尽保。切实安排好困难群众生产生活问题。

　　全力维护社会和谐稳定。深化“平安固原”创建活动，完善社会治安防控体系，加强社会治安综合治理，严厉打击黑恶势力、“两抢一盗”、民间非法借贷、传销等违法犯罪行为，切实提高群众的安全感。严密防范和打击各种敌对势力的渗透破坏活动，切实维护国家安全。健全完善安全生产责任体系，加大隐患排查治理力度，坚决遏制重特大事故的发生。加强气象、地震、地质灾害的监测预警、防灾减灾及应急处置工作，完善应急救援体系，切实保障人民群众生命财产安全。整顿规范市场经济秩序，加强价格监管，切实抓好“菜篮子”工程，满足城乡居民需求。进一步加强军政军民团结，深入推进双拥共建。依法管理宗教事务，切实加强民族团结。加强基层政权和民主政治建设。

　　八、加快转变政府职能，提升行政效能和执行力

　　认真贯彻落实市委二届九次全体会议精神，不断提高各级干部推动科学发展能力、维护社会稳定能力和解决民生突出问题能力，自觉担当起推动固原科学发展、跨越发展的历史使命。

　　强化学习，提升素质。进一步完善理论学习制度，创新学习载体，营造学习氛围。坚持组织导学、领导带学、先进引学、实践促学“四学机制”，着力解决学习的推动力发展力带动力。加大公务员和各级干部的教育培训力度，提升干部队伍综合素质。坚持理论联系实际，学以致用，不断提高领导科学发展的能力和水平。

　　深化改革，扩大开放。积极稳妥推进土地经营权流转、集体林权制度改革和生态补偿试点，为农村发展提供强大动力。积极稳妥地推进事业单位改革。加快公共财政体系建设，优化财政支出结构，提升财政保障能力。深化农村金融体制改革，引导各类金融机构将业务范围向农村延伸，进一步优化和改善金融生态环境。推进户籍制度改革，放宽县城和小城镇落户条件，解决好农民工在城镇就业和生活问题。不断扩大对外开放，建设内陆开放型城市。

　　依法行政，科学决策。全面落实行政执法责任制、执法过错追究制，严格按制度和程序办事，规范行政行为，提高依法行政水平。完善专家咨询、决策听证、新闻发布等制度，自觉接受人大依法监督、政协民主监督、社会公众监督和舆论监督，及时认真办理人大代表议案、建议和政协委员提案、建议，推进决策科学化、民主化、法制化。

　　激情干事，提高效能。面对新形势新任务，进一步提振信心，创先争优，始终保持昂扬向上、奋发有为的工作激情，始终保持求真务实、艰苦创业的工作作风，始终保持奋起直追、只争朝夕的精神状态，把精力放在工作落实上，把功夫下在推动发展上，把心思放在改善民生上，切实把各项工作抓出成效。进一步转变政府职能，减少和规范行政审批事项，办好政务服务中心、民生服务中心，加快电子政务建设，全面推行政务公开，不断推进管理创新，提升行政效能。

　　廉洁奉公，执政为民。认真落实党风廉政建设工作责任制，健全反腐倡廉长效机制。扎实推进政风行风建设，大力实施纪检监察为民工程、创优经济社会发展环境工程和勤廉为民工程，进一步加大对重点部门、领域、行业及与民生密切相关项目资金的监督检查，深入开展工程领域突出问题专项治理。继续推进廉政风险防范管理和勤政廉政承诺制，加强公务人员勤政廉政教育，提高政府工作人员执政为民的能力和水平。

　　各位代表!固原正处在一个新的历史起点。回顾过去，我们豪情满怀;展望未来，我们信心百倍。让我们高举中国特色社会主义伟大旗帜，在自治区党委、政府和市委的坚强领导下，深入贯彻落实科学发展观，“以不息为体，以日新为道”，大力弘扬“不到长城非好汉”的革命精神，凝聚全市人民的智慧和力量，团结一心，振奋精神，锐意进取，乘势前进，为开创“十二五”时期全市经济社会科学发展、跨越发展的新局面而努力奋斗!
